# Feasibility assessment of a low‐cost visible spectroscopy‐based prototype for monitoring polyphenol extraction in fermenting musts

**DOI:** 10.1002/jsfa.13274

**Published:** 2024-02-04

**Authors:** Gianmarco Alfieri, Margherita Modesti, Andrea Bellincontro, Francesco Renzi, Jose Luis Aleixandre‐Tudo

**Affiliations:** ^1^ Department for Innovation of Biological, Agrofood and Forest Systems (DIBAF) University of Tuscia Viterbo Italy; ^2^ Nature 4.0 Società Benefit Srl Viterbo Italy; ^3^ Departamento de Tecnología de Alimentos, Instituto de Ingeniería de Alimentos (Food‐UPV) Universidad Politécnica de Valencia Valencia Spain

**Keywords:** polyphenols, visible (VIS) spectrophotometer, tannins, anthocyanins, non‐destructive analysis

## Abstract

**BACKGROUND:**

Polyphenols have long been used to evaluate grape and wine quality and it is necessary to measure them throughout various winemaking stages. They are currently assessed predominantly through analytical methods, which are characterized by time‐consuming procedures and environmentally harmful practices. Non‐destructive spectroscopy‐based devices offer an alternative but they tend to be costly and not readily accessible for smaller wineries. This study introduces the initial steps in employing a portable, user‐friendly, and cost‐effective visible (VIS) spectrophotometer prototype for direct polyphenol measurement during winemaking.

**RESULTS:**

Grapes (cv Syrah, Bobal, and Cabernet Sauvignon) at different maturation stages were fermented with or without stems. Throughout fermentation, parameters such as color intensity, total polyphenol index, total anthocyanins, and tannins were monitored. Concurrently, VIS spectra were acquired using both the prototype and a commercial instrument. Chemometric approaches were then applied to establish correlation models between spectra and destructive analyses. The prototype models demonstrated an acceptable level of confidence for only a few parameters, indicating their lack of complete reliability at this stage.

**CONCLUSIONS:**

Visible spectroscopy is already utilized for polyphenol analysis in winemaking but the aspiration to automate the process in wineries, particularly with low‐cost devices, remains unrealized. This study investigates the feasibility of a low‐cost and user‐friendly spectrophotometer. The results indicate that, in the early stages of prototype utilization, the goal is attainable but requires further development and in‐depth assessments. © 2024 The Authors. *Journal of The Science of Food and Agriculture* published by John Wiley & Sons Ltd on behalf of Society of Chemical Industry.

## INTRODUCTION

Polyphenols play an important role in the quality of red wine. On one hand, the phenolic composition of wines is responsible for their organoleptic properties (i.e., body, structure, and astringency). Polyphenols can also be used as a fingerprint for their differentiation, according to the geographical origin, variety, and vintage.^1^ On the other hand, polyphenols are known for their antioxidant properties, which play an important role in wine longevity and human health.^1^, ^2^ Hence, polyphenols exhibit beneficial effects on human health, such as anti‐inflammatory, anticarcinogenic, and cardio‐protective effects.^1^, ^2^, ^3^, ^4^ Moreover, polyphenols have been used for aiding the traceability and identification of grapes in certain quality wines, such as Brunello di Montalcino.^5^

In this context, monitoring polyphenols at different wine production stages becomes crucial for the production of high‐quality wines, given the variations in polyphenol concentration between different wines and the strong impact of vinification processes.^6^, ^7^, ^8^, ^9^, ^10^

Monitoring the extraction of these compounds during the contact period between must, skins, and seeds for red wine production is very important.^7^ To date, destructive and analytical approaches have been used for the analysis and identification of polyphenols. Traditional methods for quantifying polyphenols in wine include the Folin–Ciocâlteu assay for total phenolic content, high‐performance liquid chromatography (HPLC), liquid chromatography‐mass spectrometry (LC‐MS), and various spectrophotometric and colorimetric assays targeting specific polyphenolic groups. However, these methods have limitations – for example, lack of specificity, being labor‐intensive, destructive, generating chemical waste (such as the heavy metals present in the Folin– Ciocâlteu reagent, hydrochloric acid, aluminum chloride, and so on),^10^ requiring expensive equipment, and having limited throughput. Analytical approaches also require time, a laboratory, expensive instruments, and trained staff. In recent years, spectroscopy has been recognized as one of the most interesting tools for the non‐destructive analysis of quality parameters and polyphenols in wine.^11^, ^12^, ^13^, ^14^ Spectroscopy has established itself firmly as a vital analytical tool in the evaluation of wine. Within the winemaking industry, these techniques have been adopted eagerly for the routine examination of chemical constituents, encompassing crucial parameters such as pH, sugar content, and ethanol concentration.^15^ The relentless progress in instrumentation has offered the wine industry a diverse array of accessible and adaptable measurement tools. Recent and pioneering applications of these spectroscopic techniques extend to the quantification of wine volatiles,^16^ phenolic compounds, and antioxidants,^17^, ^18^, ^19^, ^20^ as well as the assessment of sensory attributes.^21^ Visible near‐infrared (VIS‐NIR) technology has been used in oenology to measure color by means of color intensity;^22^, ^23^ alcohol content in bottled wine;^24^ tartaric acid, acetic acid, malic acid and lactic acid;^25^ for sugar monitoring,^18^ to measure polyphenol families and individual phenolics,^26^ or to determine the geographical origins of different wines.^27^, ^28^ Visible (VIS) spectroscopy, as a non‐destructive technology, must be supported by multivariate data analysis and chemometrics.^29^, ^30^

Spectrophotometric‐based methods have gained popularity due to their perceived benefits, such as cost‐effectiveness and straightforward experimental procedures but they also come with limitations. One major drawback is their lack of specificity and reproducibility.^31^, ^32^ This is because the reagents used in the assays are not exclusively selective for the intended target substances, and other substances may absorb at the same wavelengths of interest, leading to potential interference. Moreover, they require trained staff, have an initial high cost (directly linked to the instruments), and it is impossible to move the instruments. As such, performing analysis directly in the cellar during fermentation becomes difficult. In this context, the study proposed here explores the potential of a low‐cost and portable VIS spectrometer prototype, developed by Nature 4.0 (Viterbo, Italy), for monitoring polyphenol extraction during winemaking. This prototype's affordability, user‐friendly design, rapid data acquisition, and minimal operating costs make it an attractive tool for wineries. The objective is to assess its performance in comparison with a bench‐top VIS spectrophotometer, to develop prediction calibrations, and to explore its practical suitability in winemaking.

## MATERIALS AND METHODS

### Sampling and experimental design

**Figure 1:**
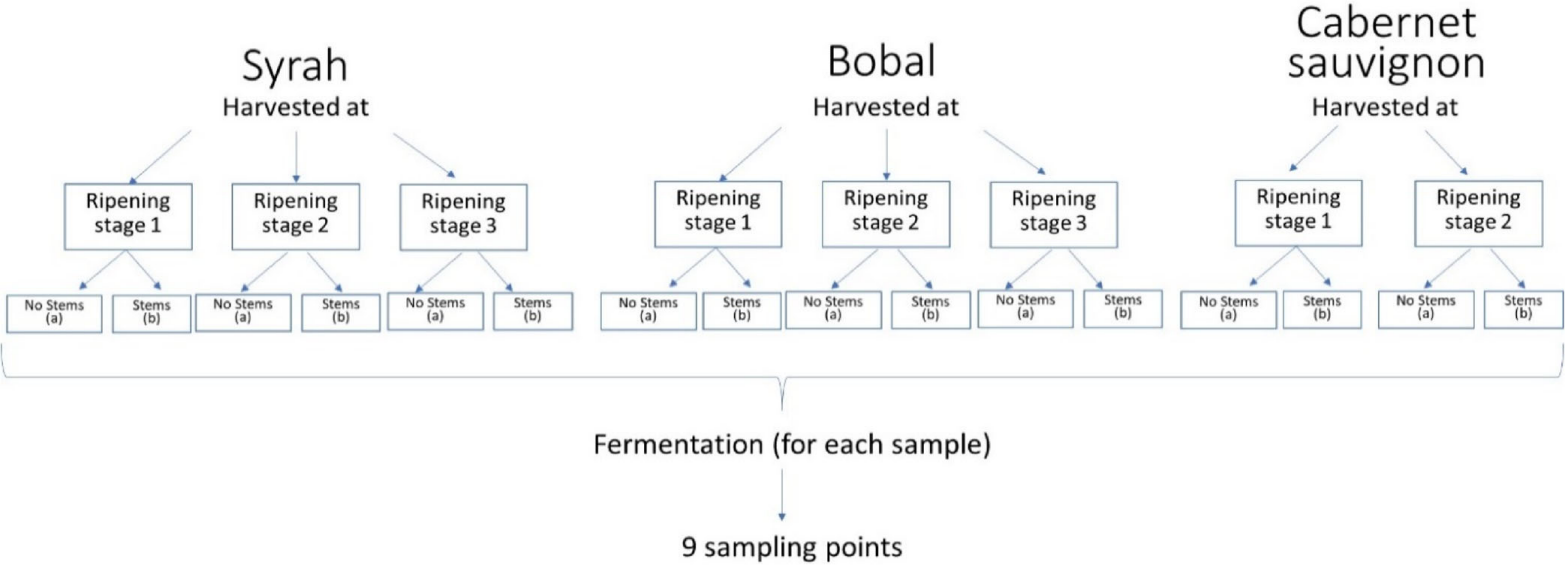
Sampling and experimental design adopted for the three grape varieties.

The grapes were harvested from the ‘Coloraos’ experimental vineyard in the municipality of Requena, Spain during the 2022 vintage. The grapes of three different varieties (*Vitis vinifera* L. cv Syrah, Bobal, and Cabernet Sauvignon) were harvested between September 15 and September 29 at three distinct sampling points, except for Cabernet Sauvignon, which was sampled twice (Fig. 1). Grapes were collected at different degrees of ripeness and fermented separately. For each fermentation, part of the grapes was crushed and destemmed (‘a’ series), and part of the grapes were crushed and fermented including stems (‘b’ series). To summarize, six different fermentations were carried out for Syrah and Bobal, and four different fermentations for Cabernet Sauvignon (Fig. 1), presenting different polyphenol concentrations.

### Winemaking

Grape bunches (~5 kg per sample) were crushed and de‐stemmed (no de‐stemming was carried out for the ‘b’ series) in a 10 L glass jar and inoculated with 30 mg/L of commercial yeasts (Zymaflore RX60 red wine yeast, Renteria, Spain), and 0.03 g/kg of sulfur dioxide was added as an 8% solution of potassium metabisulfite. To ensure variability in terms of phenolic extraction, destemmed grapes were fermented on skins at controlled temperature and relative humidity (18 °C and 80% HR), and the fermentation with stems was performed at room temperature. Must/wine samples were taken daily for a total of 9 days, centrifuged, and filtered at 0.45 μm.

### Analyses

The collected fermenting musts were used to determine different phenolic parameters. Specifically, the total polyphenol index (TPI) was obtained after diluting the wine 50 times with distilled water and by reading the absorbance at 280 nm with 10 mm quartz cuvettes.^33^ Distilled water was used as a reference blank. Total and bleachable anthocyanins were determined using the sodium hydrogen sulfite bleaching method, even though only total anthocyanins were included in the model building up.^34^ To determine color intensity (CI), wine samples were spectrophotometric assayed at three wavelengths (420, 520, and 620 nm) and the results were added to compute the index values.^35^ The determination of tannins was performed by using the methyl cellulose precipitation (MCP) method.^36^, ^37^ Three replicates were performed per sample for each analysis, except for the measurement of color intensity. For the measurement of the different analytical parameters a commercial ultraviolet visible light spectrophotometer (Jasco 730 UV–visible), working in the 200–705 nm range, with 2 nm wavelength increments and 1 mm quartz cuvette, was used.

### Spectral detections through the prototype device

**Figure 2:**
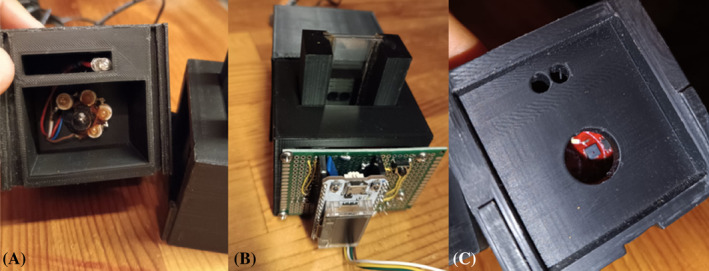
(A) The low‐cost lamp of the visible (VIS) spectrophotometer. (B) The 1 mm cuvette housing and the esp32 microcontroller. (C) The three sensors with optical filters.

The low‐cost prototype (Fig. 2) was developed in the laboratories of Tuscia University in cooperation with Nature 4.0 (Viterbo, Italy). The device is a similar model to the one described by Taglieri et al.,^37^ but presenting a spectral acquisition based on eight wavelengths between 460 and 680 nm with deviation in each wavelength of ~25–30 nm.^38^ The low‐cost prototype was developed with three multi‐channel sensors each with a specific wavelength segment. The three sensors had optical filters for a read‐out with a resolution of ~20 nm. The light source is a low‐cost halogen lamp implemented by two low‐cost LEDs, while the structure of the instrument is molded in a rigid plastic case. The AS7265x chipset consists of three sensor devices that is, AS72651 (with master capability), AS72652 and AS72653. The combination of these three sensors forms the multispectral sensor chip‐set with different channels. The multispectral sensors can be used for spectral identification in a range from VIS to NIR. All of the three sensor devices have independent on‐device optical filters whose spectral response is defined in a range from 410 to 680 nm. Using the AS7265x chipset requires the use of firmware. The components AS72651, AS72652, and AS72653 are pre‐calibrated with a specific light source. Each AS7265x device has two integrated LED drivers with programmable current and can be timed for electronic shutter applications. All three of these six‐channel devices use a conversion process implemented through two sets of photodiodes in each device. In the first set (Bank 1), data are gathered from four out of the six photodiodes, with two registers zeroed. The second set (Bank 2) gathers data from a different set of four photodiodes, with two different registers zeroed. Spectral conversion necessitates setting the integration time (IT in milliseconds) to complete the process. If both photodiode banks are needed for the conversion, the second bank requires an additional integration time in milliseconds. The minimum integration time for a single bank conversion is 2.8 milliseconds. If data from all six photodiodes are necessary, the device must undergo two full conversions (2 × integration time). The device integrates Gaussian filters into standard complementary metal oxide semiconductor (CMOS) silicon via nano‐optic deposited interference filter technology in land grid array (LGA) packages, which also provide built‐in apertures to control the light entering the sensor array. All optical characteristics are designed for optimal performance under diffused light conditions. However, if a point light source or collimated light is used on the sensor, it is necessary to cover the sensor opening with a Lambertian diffuser that exhibits achromatic characteristics. The instrument is designed with ‘Arduino’ software interface and connected to a laptop. Spectra of must/wine samples were detected using a 1 mm quartz cuvette. For each sample, the prototype performed 10 readings and provided an average spectrum.

### Data analysis

The absorbance values obtained through both the commercial and prototype spectrophotometers were used for computing linear regression models using principal component regression (PCR). The VIS detections were set as dependent variables (response variables), and destructive analyses (i.e., color intensity, total polyphenol index, anthocyanins, and tannins) as independent ones (predictor variables). Both datasets were auto‐scaled before to be addressed to chemometric computations, where venetian blind validation (blind thickness = 1; number of data split = 10) was used as cross‐validation method. Principal component regressions (PCRs) were computed for testing the correlation between detected spectra and each single analytical parameter. Determination index (R^2^) in calibration (Cal), and cross‐validation (CV), root mean standard error in calibration (RMSEC), and cross‐validation (RMSECV), and relative percent difference (RPD, SD/RESEC) were calculated and reported as indexes of statistical performance. The multivariate analyses were performed by Matlab R2013a (MathWorks, Natick, MA, USA) and PLS Toolbox (Eigenvector Research, Inc., Manson, WA, USA).

## RESULTS

### Monitoring of polyphenol extraction by analytical method

The analytical parameters (i.e., color intensity, total polyphenol index, anthocyanins, and tannins) measured in the different wines are shown in Supporting Information, Tables S1 (Syrah), S2 (Bobal), and S3 (Cabernet Sauvignon). The b‐series samples (those vinified with stalks) of any grape variety showed a significant increase in tannin concentration. This was certainly due to the extraction of these compounds from the stalks following the increase of ethanol in the medium.

Tannins for Bobal, in the absence of stalks, rose from 1000 mg/L at the third fermentation day to 2458.51 mg/L at the last fermentation day, peaking at 2535.10 mg/L at the eighth fermentation day. For Bobal, both the color intensity and anthocyanin content are slightly higher in the series without stems. This can be attributed to the ‘sponge’ effect of the stems, which tends to color the stems and therefore subtracts color from the must.^35^ The concentration of anthocyanins rose in the a‐series from 484.66 mg/L for sample 1, 400.71 mg/L for sample 2 and 554.97 mg/L for sample 3 on the first day of fermentation, to 354.99, 490.53, and 458.19 mg/L respectively. The b‐series, on the other hand, rose from 861.57 mg/L in sample 1, 751.84 mg/L in sample 2 and 897.44 mg/L for sample 3 on the first day of fermentation to 588.35, 649.64, and 835.76 mg/L, respectively. The TPI gradually increased with higher increases in the last days of fermentation; obviously the b‐series samples reached higher average TPI levels than the a‐series, vinified without stalks. On the last fermentation day, however, a decrease in the total polyphenol value was observed in both sets of samples. The TPI followed an upward trend, rising in the a‐series from 14.46 in sample 1, 5.93 in sample 2 and 23.79 in sample 3 on the first day of fermentation to 62.97, 47.12 and 80.3, respectively. The b‐series, on the other hand, rose from 19.15 in sample 1, 27.9 in sample 2 and 30.77 in sample 3 on the first day of fermentation to 66.01, 71.13, and 96.66 in samples 1, 2 and 3, respectively.

As far as Syrah is concerned, the tannin concentration in destemmed vinification doubled from the first to the last day of fermentation, and it increased by about 2.5 to 3 times for musts fermented with stems. The starting tannin content for the a‐series was 439.3 mg/L for sample 1, 390.4 mg/L for sample 2 and 309.2 mg/L for sample 3 on the first day of fermentation. At the end of the fermentation it stood at 1010.2, 1049.6, and 1289.8 mg/L respectively. The b‐series, on the other hand, rose from 1098.8 mg/L in sample 1, 1177.6 mg/L in sample 2 and 766.4 mg/L for sample 3 on the first day of fermentation to 2419, 3233.1, and 2530.4 mg/L, respectively. The color intensity between the first and last day of sampling remained very similar, although some peaks were observed at 2.74 for sampling 6 of series 2b and 2.70 for 3b, showing how incorrect oenological activities can take place and color intensity can be lost if this parameter is not monitored. Anthocyanins doubled its content in both series, with a slight overall decrease on last fermentation day (day 9). The TPI increased during fermentation and appears to be more stable than in Bobal.

For Cabernet Sauvignon, a large increase in tannin concentration was observed in both series. However, in the de‐stemmed series the increase was higher, while for the b‐series a smaller increase was observed, but the starting concentration was higher, again due to the presence of the stalks. The starting tannins for the a‐series were 230.1 mg/L for sample 1 and 558.2 mg/L for sample 2 on the first day of fermentation, and then stood at 1228.7 and 1295.8 mg/L, respectively. The b series, on the other hand, rose from 573 mg/L in sample 1 and 1191.5 mg/L in sample 2 on the first day of fermentation to 1991.4 and 2370.2 mg/L, respectively. Cabernet wines’ color intensity and anthocyanins were the least stable, reaching slightly higher values at the end of fermentation than those found on the first fermentation day. The TPI followed an upward trend throughout the test, rising in the a‐series from 7.01 for sample 1 and 29.61 for sample 2 on the first day of fermentation, and then stood at 14.05 and 32.92, respectively. The b‐series, on the other hand, rose from 61.4 in sample 1 and 63.26 in sample 2 on the first day of fermentation to 73.48 and 105.18, respectively in samples 1 and 2. The Cabernet Sauvignon grapes were the ones with the lowest visual ripeness in comparison with the other varieties.

### Monitoring polyphenol extraction with a commercial VIS spectrophotometer

**Figure 3:**
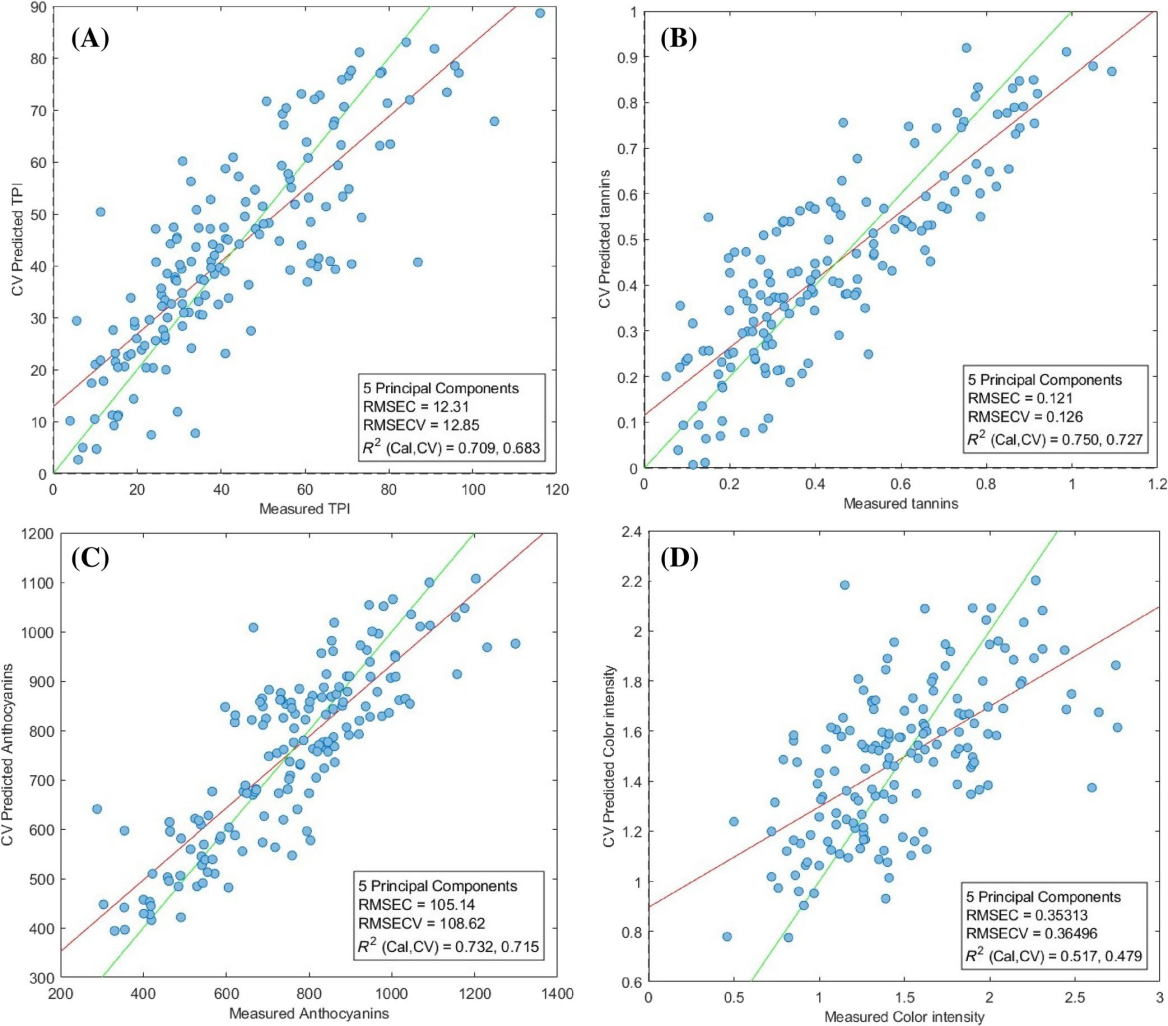
Scatter plots referred to the PCR modelings (observed vs. predicted data), based on the visible spectra detected by the commercial device, for the following parameters: total polyphenols index (I_280_, arbitrary unit) (A), tannins (mg/L) (B), anthocyanins (mg/L) (C), and color intensity (arbitrary unit) (D). RMSEC, root mean standard error in calibration; RMSECV, root mean standard error in cross‐validation; *R* ^2^Cal, determination coefficient in calibration; *R* ^2^CV, determination coefficient in cross‐validation.

To compare the performance of the two spectrophotometers in predicting the different analytical parameters, PCR models were built using both instruments' VIS information. As far as the commercial instrument was concerned, five principal components were selected to reach a cumulative explained variability of 96%. The obtained determination coefficients (*R* ^2^) values were quite positive. Specifically, for TPI *R* ^2^ values of 0.709 and 0.683 were obtained in calibration (Cal) and cross‐validation (CV) respectively. (Fig. 3A). A good prediction aptitude was also observed for tannins (0.750 in calibration and 0.727 in cross‐validation) (Fig. 3B), and anthocyanins (0.732 in calibration and 0.715 in cross‐validation) (Fig. 3C). On the other hand, the worst performance was observed for the color density model (*R* ^2^ of 0.431 and 0.387 for calibration and cross‐validation respectively) (Fig. 3D). Model details are shown in Table 1.

**Table 1:** Summary table of both descriptive and multivariate indexes for PCR models reported in Figs  3 and 4

Statistical indexes		TPI	Tannins	Anthocyanins	CI
Mean		42.5	0.4	750	1.4
SD		22.8	0.24	203	0.4
*n*		160	160	160	160
Min.		4	0.05	288	0.4
Max.		116	1	1298	2.7
RMSEC	Commercial	12.31	0.12	105.1	0.35
	Prototype	13.88	0.15	114	0.36
RMSECV	Commercial	12.85	0.12	106.62	0.36
	Prototype	14.07	0.16	117.45	0.37
RPD	Commercial	1.85	2	1.93	1.31
	Prototype	1.64	1.6	1.78	1.27
Error (%)	Commercial	30.24	30.00	14.22	25.71
	Prototype	61.71	66.67	57.86	92.50

CI, color intensity; Error (%) = (RMSECV/mean) × 100; SD, standard deviation; RMSEC, root mean standard error in calibration; RMSECV, root mean standard error in cross‐validation; RPD, relative percent difference (SD/RMSEC); TPI, total polyphenol index.

### Monitoring polyphenol extraction by prototype VIS spectrophotometer

**Figure 4:**
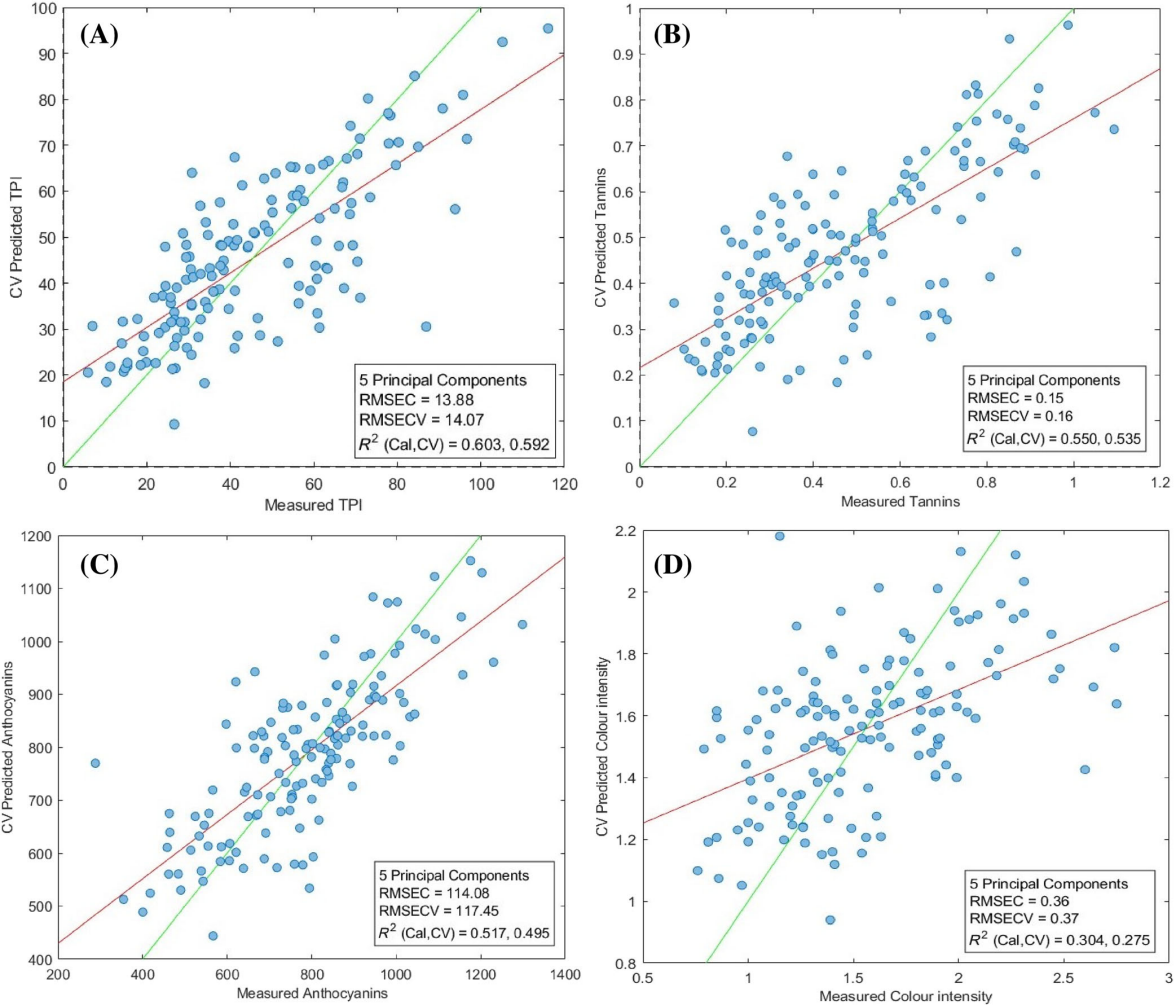
Scatter plots referred to the PCR modelings (observed vs. predicted data), based on the visible spectra detected by the prototype, for the following parameters: total polyphenols index (I_280_, arbitrary unit) (A), tannins (mg/L) (B), anthocyanins (mg/L) (C), and color intensity (arbitrary unit) (D). RMSEC, root mean standard error in calibration; RMSECV, root mean standard error in cross‐validation; *R* ^2^Cal, determination coefficient in calibration; *R* ^2^CV, determination coefficient in cross‐validation.

As previously mentioned, to verify the differences in terms of performance in predicting the different parameters, regressive models were built with the prototype‐device‐acquired spectra by using the same criteria for the PCR modeling as were used for the commercial device. In this case, five principal components explained 79.65% of the variability. The *R* ^2^ coefficients in prediction were lower than the *R* ^2^ coefficients relative to the models obtained from the commercial spectrophotometer. Specifically, an *R* ^2^ of 0.603 and 0.592 was obtained in calibration and cross‐validation for TPI (Fig. 4A), 0.550 and 0.535 for tannins (Fig. 4B), and 0.517 and 0.495 for anthocyanins (Fig. 4C). As observed for the commercial spectrophotometer, the prototype shows the worst performance in predicting the color intensity, with lower correlation coefficients (*R* ^2^ 0.304 and 0.275, respectively). Model details are shown in Table 1.

## DISCUSSION

As described above, the current study focuses on the advantages and potential applications of VIS spectroscopy, with particular attention to polyphenol and color analysis in wine during fermentation. Accurate predictive performance was achieved by the commercial benchtop VIS spectrophotometer model, confirming that VIS spectroscopy can be a substitute for wet chemistry, substantially helping to reduce the use of chemical reagents and greatly limiting the environmental impact of oenological analyses as well as the time required for performing them. Moreover, the fact that visible spectral data were used to predict UV absorbing compounds such as tannins and the majority of the phenolics of the TPI adds value to this VIS spectroscopy application. The VIS spectrophotometric methods offer a unique set of benefits and numerous techniques have been suggested for the measurement of phenolic compounds.^39^, ^40^, ^41^ Nevertheless, despite the widespread adoption of spectrophotometric approaches, primarily because of their recognized advantages such as cost‐effectiveness and simplified experimental procedures, they do exhibit notable limitations. For instance, an important drawback is their lack of specificity and reproducibility.^33^ This is because the reagents used in the assays are not exclusively selective for the intended target substances, and other substances may absorb at the same wavelengths of interest, leading to potential interference. The use of spectroscopy calibrations overcomes these limitations.

Even though the predicting aptitude of the spectral prototype device tested here was lower, a significant consideration that deserves to be highlighted is the strong difference in terms of cost between the two devices. The prototype VIS spectrometer made by Nature 4.0 has a significantly lower cost: the production cost which includes lamp, sensors, electrical and structural components is under 50 euros. Predictive models computed on data from the VIS prototype coupled with destructive analyses generally perform positively, even though they always present lower correlations than those achieved employing commercial instrumentation. The best values were measured for the prediction of the total polyphenol index with comparable cross‐validation errors of 12.85 and 14.07 index values for the commercial and protype instruments, respectively. With respect to the models built for the prediction of total anthocyanins, the observed difference on the computed cross‐validation error was 108.62 and 117.45 mg/L of anthocyanins for the commercial and prototype spectrometers, respectively. These differences in prediction errors show the potential of the prototype compared with the commercial device.

The results showed that the predictive efficacy of PCR models generated using spectra derived from the VIS prototype device slightly underperformed those produced with the commercial instrument. The lower performance of the prototype is clearly apparent from the percentage errors in prediction (Table 1), where the observed error rates consistently exhibited double the values observed with the commercial spectrophotometer. This unequivocally underscores the prototype's limitations and emphasizes the imperative of further refinement and optimization in future iterations. The differences between the predictive accuracy are likely to be due to the disparity in the number of wavelength data points employed in instrumental detection: eight for the prototype and 104 for the commercial apparatus. Moreover, the lack of an absorption wavelength at 280 nm makes the prediction of tannin content and the total polyphenol index particularly complex, as 280 nm corresponds to the peak absorption wavelength associated with this specific phenolic category. It is also important to incorporate wavelengths around 320 nm into the spectrophotometer to enhance its analytical performance. This wavelength range encompasses hydroxycinnamic acids (comprising free molecules such as caffeic, *p*‐coumaric, ferulic, and sinapic acids) and their tartaric esters, typified by caftaric acid. It also includes benzoic acids characterized by their predominant form, gallic acid.^42^ Furthermore, to ascertain the presence of specific stilbenes, the inclusion of a detection wavelength at 307 nm would be advantageous for monitoring the presence of resveratrol and its conjugated forms. Analogous to tannins, numerous polyphenolic families exhibit absorbance peaks at 280 and 365 nm (e.g., quercetin), thereby contributing to a diminished predictive performance, even within the overall polyphenol index. Tannins constitute a prominent class of phenolic compounds, exerting substantial influence on the overall polyphenolic composition of red wine.^43^, ^44^, ^45^, ^46^ Wine's color components are critical parameters contributing significantly to its sensory attributes, including color perception and astringency, and playing a crucial role in determining its antioxidant properties.^47^, ^48^ The objective measurement of wine color components is an integral part of modern winemaking and the development of new methods for quantifying pigments has become crucial.

Several studies have demonstrated the efficacy of visible spectroscopy for the prediction of phenolic content in wine.^49^, ^50^, ^51^, ^52^ Innovative methods of detection have also been developed. For instance, Gu et al. developed an UV‐visible prototype coupled with machine‐learning computation to identify red wines coming from different geographical areas of production.^53^ Moreover, a UV‐visible model combined with chemometric tools was recently used to predict the phenolic content of Pinot noir wines.^54^ However, low‐cost, easy‐to‐use and portable spectrophotometers are quite rare. Consequently, once the clear gaps and technical limitations of the prototype proposed here have been addressed, especially regarding the addition of important wavelengths, the prospect of employing such an analytical tool within winemaking operations holds significant appeal.

## CONCLUSIONS

Principal component regression models obtained throughout the spectral detections performed using the commercial VIS spectrophotometer were found to be suitable for predicting total polyphenol index, anthocyanins and tannins. This confirms that non‐destructive VIS techniques can be used as valid alternative to wet chemistry for monitoring polyphenol extraction during vinification processes. On the other hand, regression models built with a VIS prototype showed only slightly less accurate models. This is likely explainable in terms of the different reading points in the two instruments (only eight wavelengths in the prototype). As such, to increase the accuracy of the prototype, other wavelengths need to be included and, among them, certainly those referred to 280, 320, and 365 nm, which are the wavelengths of greatest interest for correlating polyphenol families. These additions could certainly bring the prototype accuracy at least to a similar level as commercial instruments. Moreover, there is a need to find an effective and reproducible sampling protocol for fermenting musts that can facilitate the measurements directly in the cellar.

## Supporting information


**Table S1.** Color intensity (CI), total polyphenol index (TPI), anthocyanins and tannins measured with analytical methods in Syrah musts at different degree of ripeness (Sample 1–9) fermented without (series a) and with (series b) stems. Values are the mean of three replicate ± standard deviation.
**Table S2.** Color intensity (CI), total polyphenol index (TPI), anthocyanins and tannins measured with analytical methods in Bobal musts at different degree of ripeness (Sample 1–9) fermented without (series a) and with (series b) stems. Values are the mean of three replicate ± standard deviation.
**Table S3.** Color intensity (CI), total polyphenol index (TPI), anthocyanins and tannins measured with analytical methods in Cabernet Sauvignon musts at different degree of ripeness (Sample 1–9) fermented without (series a) and with (series b) stems. Values are the mean of three replicate ± standard deviation.

## Data Availability

The data that support the findings of this study are available from the corresponding author upon reasonable request.
