# Supplementary material for: Feasibility assessment of a low‐cost visible spectroscopy‐based prototype for monitoring polyphenol extraction in fermenting musts
Source: J Sci Food Agric. 2024 Feb 4;105(3):1456–64. doi: 10.1002/jsfa.13274 (PMC11726600; doi:10.1002/jsfa.13274)
Supplement: Supplementary file 1 — Table S1. Color intensity (CI), total polyphenol index (TPI), anthocyanins and tannins measured with analytical methods in Syrah musts at different degree of ripeness (Sample 1–9) fermented without (series a) and with (series b) stems. Values are the mean of three replicate ± standard deviation. Table S2. Color intensity (CI), total polyphenol index (TPI), anthocyanins and tannins measured with analytical methods in Bobal musts at different degree of ripeness (Sample 1–9) fermented without (series a) and with (series b) stems. Values are the mean of three replicate ± standard deviation. Table S3. Color intensity (CI), total polyphenol index (TPI), anthocyanins and tannins measured with analytical methods in Cabernet Sauvignon musts at different degree of ripeness (Sample 1–9) fermented without (series a) and with (series b) stems. Values are the mean of three replicate ± standard deviation. [file JSFA-105-1456-s001.docx]

Table S1 Color intensity (CI), total polyphenol index (TPI), anthocyanins and tannins measured with analytical methods in Syrah musts at different degree of ripeness (Sample 1 to 9) fermented without (series a) and with (series b) stems. Values are the mean of three replicate ± standard deviation.

|  |  | **CI** | **TPI** | **Anthocyanins mg/L** | **Tannins mg/L** |
| --- | --- | --- | --- | --- | --- |
| **Sampling day 1** | **syrah1a** | 1.4 | 10.31 ± 0.83 | 543.46 ± 18.31 | 439.3 ± 17.9 |
|  | **syrah2a** | 1.26 | 15.33 ± 0.89 | 605.46 ± 23.33 | 390.4 ± 5.1 |
|  | **syrah3a** | 1.27 | 14.25 ± 0.79 | 538.78 ± 5.51 | 309.2 ± 6 |
|  | **syrah1b** | 1.39 | 26.85 ± 0.43 | 566.91 ± 3.71 | 1098.8 ± 36.5 |
|  | **syrah2b** | 1.21 | 36.20 ± 1.29 | 464.41 ± 1.3 | 1177.6 ± 20.8 |
|  | **syrah3b** | 1.38 | 27.22 ± 1.07 | 621.29 ± 18.38 | 766.4 ± 17.5 |
| **Sampling day 2** | **syrah1a** | 1.4 | 19.85 ± 0.05 | 702.84 ± 15.16 | 637.4 ± 52.1 |
|  | **syrah2a** | 1.41 | 24.48 ± 0.02 | 722.75 ± 27.47 | 630.1 ± 14.3 |
|  | **syrah3a** | 1.43 | 25.72 ± 0.02 | 669.24 ± 20.11 | 810.8 ± 18 |
|  | **syrah1b** | 1.39 | 51.39 ± 0.01 | 763.96 ± 8.29 | 2187.8 ± 108 |
|  | **syrah2b** | 1.96 | 54.63 ± 0.04 | 891.76 ± 3.65 | 2228.3 ± 273.2 |
|  | **syrah3b** | 1.54 | 44.16 ± 0.04 | 791.22 ± 18.99 | 1498.0 ± 112.3 |
| **Sampling day 3** | **syrah1a** | 1.58 | 19.33 ± 1.6 | 738.59 ± 11.38 | 468.2 ± 57.6 |
|  | **syrah2a** | 1.61 | 24.40 ± 1.33 | 766.28 ± 7.36 | 667.1 ± 41 |
|  | **syrah3a** | 1.61 | 26.14 ± 1.14 | 803.69 ± 45.91 | 627.5 ± 50.8 |
|  | **syrah1b** | 1.62 | 60.86 ± 1.03 | 799.36 ± 1.55 | 2310.9 ± 170 |
|  | **syrah2b** | 2 | 63.53 ± 3.61 | 1007.91 ± 22.89 | 2441.5 ± 196.9 |
|  | **syrah3b** | 1.74 | 50.84 ± 0.16 | 829.85 ± 9.16 | 1508.3 ± 89.9 |
| **Sampling day 4** | **syrah1a** | 1.72 | 22.92 ± 0.13 | 688.49 ± 9.22 | 817.7 ± 33.4 |
|  | **syrah2a** | 1.32 | 28.69 ± 1.17 | 776.74 ± 28.09 | 870.7 ± 44.4 |
|  | **syrah3a** | 1.54 | 29.42 ± 0.99 | 763.13 ± 33.6 | 756.0 ± 58 |
|  | **syrah1b** | 1.67 | 56.38 ± 2.36 | 841.93 ± 2.35 | 2269.9 ± 236 |
|  | **syrah2b** | 2.05 | 79.66 ± 5.61 | 939.75 ± 0.37 | 2441.5 ± 81.4 |
|  | **syrah3b** | 1.77 | 55.53 ± 5.36 | 924.13 ± 3.03 | 2012.5 ± 117.8 |
| **Sampling day 5** | **syrah1a** | 1.82 | 25.89 ± 0.21 | 840.66 ± 6.5 | 891.7 ± 51.8 |
|  | **syrah2a** | 1.84 | 34.67 ± 0.81 | 882.22 ± 31.37 | 621.3 ± 5 |
|  | **syrah3a** | 1.82 | 34.14 ± 1.68 | 808.68 ± 12 | 415.4 ± 18.1 |
|  | **syrah1b** | 1.87 | 61.35 ± 0.2 | 896.13 ± 2.29 | 2158.2 ± 143 |
|  | **syrah2b** | 2.31 | 78.32 ± 1.82 | 1002.40 ± 3.22 | 2774.1 ± 146 |
|  | **syrah3b** | 2.18 | 66.77 ± 5.23 | 946.97 ± 24.69 | 2059.8 ± 152.8 |
| **Sampling day 6** | **syrah1a** | 1.9 | 28.16 ± 1.98 | 809.59 ± 36.81 | 808.0 ± 114.4 |
|  | **syrah2a** | 1.9 | 38.39 ± 0.26 | 859.12 ± 14.17 | 1106.3 ± 43.6 |
|  | **syrah3a** | 1.99 | 45.85 ± 7.34 | 866.21 ± 43.37 | 1037.2 ± 79.2 |
|  | **syrah1b** | 2.14 | 60.39 ± 2.51 | 967.79 ± 35.21 | 2286.3 ± 134.5 |
|  | **syrah2b** | 2.74 | 84.99 ± 0.08 | 1229.94 ± 11.2 | 2872.7 ± 147.1 |
|  | **syrah3b** | 2.44 | 68.78 ± 1.41 | 1092.39 ± 22.83 | 2390.9 ± 174.5 |
| **Sampling day 7** | **syrah1a** | 1.67 | 34.65 ± 2.48 | 824.38± 22.34 | 951.8 ± 57.2 |
|  | **syrah2a** | 1.81 | 37.53 ± 3.09 | 948.33 ± 10.15 | 993.1 ± 30.5 |
|  | **syrah3a** | 1.67 | 55.98 ± 2.30 | 858.16 ± 3.03 | 1174.8 ± 54.3 |
|  | **syrah1b** | 1.88 | 60.74 ± 0.02 | 1009.31 ± 72.39 | 2639.1 ± 60.5 |
|  | **syrah2b** | 2.27 | 84.14 ± 3.09 | 1090.78 ± 8.91 | 3007.4 ± 23.4 |
|  | **syrah3b** | 1.98 | 71.02 ± 2.98 | 1046.50 ± 4.33 | 2828.5 ± 7.4 |
| **Sampling day 8** | **syrah1a** | 1.61 | 32.91 ± 1.78 | 892.89 ± 48.2 | 933.0 ± 9.4 |
|  | **syrah2a** | 2.09 | 41.04 ± 0.04 | 1068.46 ± 10.15 | 1095.8 ± 85.2 |
|  | **syrah3a** | 1.66 | 32.82 ± 0.62 | 1008.22 ± 3.4 | 1049.1 ± 23.5 |
|  | **syrah1b** | 1.74 | 69.33 ± 0.05 | 952.26 ± 31.18 | 2839.7 ± 11.4 |
|  | **syrah2b** | 2.2 | 90.86 ± 0.06 | 1153.78 ± 1.61 | 2977.7 ± 18.9 |
|  | **syrah3b** | 1.9 | 72.99 ± 0.05 | 979.96 ± 4.15 | 2549.0 ± 0.5 |
| **Sampling day 9** | **syrah1a** | 1.41 | 56.44 ± 0.72 | 758.23 ± 46.71 | 1010.2 ± 38.2 |
|  | **syrah2a** | 1.62 | 67.24 ± 0.59 | 993.13 ± 1.98 | 1049.6 ± 24.1 |
|  | **syrah3a** | 1.44 | 30.84 ± 0.41 | 854.83 ± 21.1 | 1289.8 ± 13 |
|  | **syrah1b** | 1.4 | 93.85 ± 0.96 | 860.91 ± 3.22 | 2419.5 ± 61.5 |
|  | **syrah2b** | 1.15 | 116.10 ± 1.55 | 1202.82 ± 20.48 | 3233.1 ± 199.4 |
|  | **syrah3b** | 1.62 | 95.74 ± 1.37 | 944.96 ± 11.57 | 2530.4 ± 73.4 |

Table S2 Color intensity (CI), total polyphenol index (TPI), anthocyanins and tannins measured with analytical methods in Bobal musts at different degree of ripeness (Sample 1 to 9) fermented without (series a) and with (series b) stems. Values are the mean of three replicate ± standard deviation.

|  |  | **CI** | **TPI** | **Anthocyanins mg/L** | **Tannins mg/L** |
| --- | --- | --- | --- | --- | --- |
| **Sampling day 1** | **bobal1a** | 1.17 | 14.46 ± 1.16 | 484.66 ± 17.08 | 447.05 ± 7.09 |
|  | **bobal2a** | 0.97 | 5.93± 0.06 | 400.71 ± 9.71 | 347 ± 12.47 |
|  | **bobal3a** | 1 | 23.79 ± 1.44 | 554.97 ± 1.42 | 1000 ± 62.21 |
|  | **bobal1b** | 0.86 | 19.15 ± 0.21 | 354.99 ± 7.8 | 907.99 ± 16.21 |
|  | **bobal2b** | 1.23 | 27.9 ± 0.1 | 490.53 ± 2.6 | 886.5 ± 55.58 |
|  | **bobal3b** | 0.95 | 30.77± 0.05 | 458.19 ± 3.16 | 1677.02 ± 14.68 |
| **Sampling day 2** | **bobal1a** | 1.27 | 24.39 ± 0.07 | 664.43 ± 15.03 | 810.14 ± 25.26 |
|  | **bobal2a** | 1 | 11.24 ± 0.01 | 566.13 ± 19.8 | 543.14 ± 14.38 |
|  | **bobal3a** | 1.05 | 38.41 ± 0.03 | 794.72 ± 19.49 | 1701.6 ± 27.18 |
|  | **bobal1b** | 0.74 | 30.72 ± 0.01 | 491.05 ± 5.82 | 1248.82 ± 10.17 |
|  | **bobal2b** | 1.27 | 29.03 ± 0-04 | 620.81 ± 36.31 | 1475.04 ± 99.26 |
|  | **bobal3b** | 1.1 | 54.98 ± 0.02 | 661.68 ± 20.54 | 2699.5 ± 24.61 |
| **Sampling day 3** | **bobal1a** | 1.35 | 25.79 ± 2.03 | 759.24 ± 10.77 | 812.78 ± 12.55 |
|  | **bobal2a** | 1.1 | 14.82 ± 3.25 | 606.46 ± 0.99 | 569.85 ± 34.67 |
|  | **bobal3a** | 1.2 | 40.72 ± 1.51 | 771.93 ± 14.48 | 1743.69 ± 32.55 |
|  | **bobal1b** | 0.85 | 29.07 ± 0.23 | 513.84 ± 2.66 | 1395.92 ± 59.1 |
|  | **bobal2b** | 1.41 | 30.83 ± 3.14 | 672.39 ± 7.86 | 1523.75 ± 108.99 |
|  | **bobal3b** | 1.31 | 59.13 ± 2.49 | 742.92 ± 35.08 | 2816.2 ± 104.82 |
| **Sampling day 4** | **bobal1a** | 1.54 | 26.65 ± 0.51 | 638.88 ± 15.16 | 915.04 ± 42.25 |
|  | **bobal2a** | 1.26 | 15.37 ± 1.13 | 584.15 ± 17.82 | 623.91 ± 21.71 |
|  | **bobal3a** | 1.44 | 41.01 ± 3.1 | 778.53 ± 24.69 | 1967.27 ± 37.99 |
|  | **bobal1b** | 1.02 | 34.02 ± 0.22 | 463.05 ± 4.08 | 1415.43 ± 18.14 |
|  | **bobal2b** | 1.49 | 32.34 ± 1.52 | 586.43 ± 1.48 | 1597.02 ± 129.91 |
|  | **bobal3b** | 1.31 | 62.27 ± 3.71 | 731.72 ± 14.42 | 2868.32 ± 45.62 |
| **Sampling day 5** | **bobal1a** | 1.69 | 29.39 ± 1.67 | 862.09 ± 4.02 | 976.53 ± 76.64 |
|  | **bobal2a** | 1.25 | 18.53 ± 0.11 | 691.29 ± 6.37 | 657.41 ± 32.06 |
|  | **bobal3a** | 1.44 | 48.24 ± 3.22 | 840.04 ± 8.72 | 2002.09 ± 90.61 |
|  | **bobal1b** | 1.16 | 37.42 ± 1.57 | 556.85 ± 1.24 | 1413.22 ± 99.79 |
|  | **bobal2b** | 1.81 | 41.77 ± 1.69 | 800.14 ± 11.08 | 1604.52 ± 49.86 |
|  | **bobal3b** | 1.63 | 67.03 ± 3.43 | 840.39 ± 24.32 | 2899.06 ± 16.86 |
| **Sampling day 6** | **bobal1a** | 1.94 | 30.77 ± 2.97 | 641.29 ± 22.29 | 1092.01 ± 67.92 |
|  | **bobal2a** | 1.63 | 22.12 ± 1.25 | 687.66 ± 10.02 | 826.89 ± 54.88 |
|  | **bobal3a** | 1.48 | 48.11 ± 4.47 | 921.33± 20.48 | 2146.23 ± 19.28 |
|  | **bobal1b** | 1.23 | 41.1 ± 1.53 | 524.78 ± 7.49 | 1686.72 ± 35.03 |
|  | **bobal2b** | 1.89 | 46.58 ± 1.4 | 726.82 ± 3.77 | 1615.98 ± 18.14 |
|  | **bobal3b** | 1.89 | 68.6 ± 1.9 | 834.18 ± 9.22 | 2986.01 ± 159.25 |
| **Sampling day 7** | **bobal1a** | 1.91 | 35.95 ± 1.2 | 836.46 ± 36.94 | 1262.92 ± 19.71 |
|  | **bobal2a** | 1.38 | 26.65 ± 0.75 | 672.7 ± 5.32 | 957.47 ± 45.08 |
|  | **bobal3a** | 1.82 | 56.74 ± 2.66 | 947.19 ± 13.86 | 2564.41 ± 20.06 |
|  | **bobal1b** | 1.27 | 59.19 ± 3.18 | 533.84 ± 5.94 | 1821.49 ± 75.46 |
|  | **bobal2b** | 2.6 | 59.64 ± 0.85 | 754.69 ± 2.72 | 1884.74 ± 52.34 |
|  | **bobal3b** | 2.08 | 70.42 ± 1.66 | 872.51 ± 7.98 | 3583.47 ± 18.06 |
| **Sampling day 8** | **bobal1a** | 1.58 | 37.63 ± 1.24 | 819.7 ± 7.42 | 1475.7 ± 68.44 |
|  | **bobal2a** | 1.39 | 42.86 ± 0.01 | 665.64 ± 30.94 | 1450.03 ± 41.42 |
|  | **bobal3a** | 1.91 | 54.45 ± 0.03 | 1032.46 ± 26.42 | 2535.1 ± 96.5 |
|  | **bobal1b** | 0.99 | 70.45 ± 0.52 | 646.06 ± 26.05 | 1745.45 ± 49.66 |
|  | **bobal2b** | 1.57 | 86.92 ± 3.33 | 816.99 ± 38.48 | 2140.57 ± 22.43 |
|  | **bobal3b** | 1.5 | 77.94 ± 8.5 | 1043.53 ± 7.18 | 3438.9 ± 24.14 |
| **Sampling day 9** | **bobal1a** | 1.47 | 62.97 ± 0.36 | 861.57 ± 5.63 | 1257.41 ± 11.81 |
|  | **bobal2a** | 1.01 | 47.12 ± 1.11 | 649.64 ± 22.95 | 727.82 ± 33.45 |
|  | **bobal3a** | 1.26 | 80.3 ± 3.03 | 897.44 ± 60.33 | 2458.51 ± 61.82 |
|  | **bobal1b** | 0.79 | 66.01 ± 1.41 | 588.35 ± 35.54 | 1618.18 ± 98.16 |
|  | **bobal2b** | 1.31 | 71.13 ± 1.58 | 751.84 ± 2.29 | 2180.83 ± 15.17 |
|  | **bobal3b** | 1.33 | 96.66 ± 1.79 | 835.76 ± 27.66 | 2459.5 ± 86.98 |

Table S3 Color intensity (CI), total polyphenol index (TPI), anthocyanins and tannins measured with analytical methods in Cabernet Sauvignon musts at different degree of ripeness (Sample 1 to 9) fermented without (series a) and with (series b) stems. Values are the mean of three replicate ± standard deviation.

|  |  | **CI** | **TPI** | **Anthocyanins mg/L** | **Tannins mg/L** |
| --- | --- | --- | --- | --- | --- |
| **Sampling day 1** | **cabernet 1a** | 0.76 | 7.01 ± 0.46 | 418.12 ± 38.92 | 230.1 ± 13.7 |
|  | **cabernet 2a** | 1.1 | 29.61 ± 0.89 | 546.04 ± 2.04 | 558.2 ± 18.1 |
|  | **cabernet1b** | 0.81 | 14.05 ± 0.20 | 461.69 ± 3.28 | 573 ± 21.7 |
|  | **cabernet 2b** | 1.56 | 32.92 ± 2.11 | 718.07 ± 3.03 | 1191.5 ± 23 |
| **Sampling day 2** | **cabernet 1a** | 0.99 | 17.77 ± 0.06 | 753.38 ± 24.01 | 569.5 ± 10.5 |
|  | **cabernet 2a** | 1.33 | 26.6 ± 0.06 | 748.56 ± 4.21 | 734.4 ± 16.1 |
|  | **cabernet1b** | 0.85 | 30.57 ± 0.01 | 696.06 ± 23.88 | 1277.1 ± 41.7 |
|  | **cabernet 2b** | 1.99 | 53.89 ± 0.02 | 920.24 ± 3.09 | 1609 ± 23.8 |
| **Sampling day 3** | **cabernet 1a** | 1.07 | 21.77 ± 0.46 | 880.51 ± 2.72 | 576.3 ± 12.9 |
|  | **cabernet 2a** | 1.41 | 27.18 ± 0.30 | 786.98 ± 21.53 | 764.2 ± 49 |
|  | **cabernet1b** | 1.04 | 30.19 ± 0.07 | 686.57 ± 7.86 | 1535.9 ±80 |
|  | **cabernet 2b** | 2.26 | 49.91 ± 4.05 | 937.04 ± 1.3 | 1742.5 ± 43.3 |
| **Sampling day 4** | **cabernet 1a** | 1.21 | 26.6 ± 0.38 | 778.97 ± 12.81 | 830.9 ± 15.1 |
|  | **cabernet 2a** | 1.55 | 29.49 ± 1.60 | 732.94 ± 3.16 | 871.3 ± 45.4 |
|  | **cabernet1b** | 1.13 | 37.65 ± 2.26 | 703.55 ± 12.68 | 1427.7 ± 35.6 |
|  | **cabernet 2b** | 2.19 | 57.61 ±4.78 | 964.86 ± 15.96 | 1905.8 ± 54.3 |
| **Sampling day 5** | **cabernet 1a** | 1.33 | 31.12 ± 2.80 | 831.21 ± 27.04 | 903.7 ± 11.9 |
|  | **cabernet 2a** | 1.8 | 35.09 ± 2.58 | 845.6 ± 19.18 | 927.9 ± 20.9 |
|  | **cabernet1b** | 1.14 | 39.58 ± 2.11 | 737.84 ± 47.32 | 1614.1 ± 60.4 |
|  | **cabernet 2b** | 2.45 | 65.02 ± 3.48 | 997.11 ± 41.95 | 2036.5 ± 19.8 |
| **Sampling day 6** | **cabernet 1a** | 1.61 | 35.59 ± 0.55 | 853.96 ± 0.31 | 1050.1 ± 73.5 |
|  | **cabernet 2a** | 1.85 | 38 ± 1.36 | 858.29 ± 8.17 | 1236.3 ± 52.7 |
|  | **cabernet1b** | 1.37 | 44.32 ± 1.02 | 730.14 ± 5.51 | 1738.7 ± 42.8 |
|  | **cabernet 2b** | 2.64 | 67.9 ± 0.69 | 1017.93 ± 40.65 | 2570.1 ± 93.3 |
| **Sampling day 7** | **cabernet 1a** | 1.25 | 40.9 ± 0.77 | 802.11 ± 41.21 | 1157.1 ± 31.1 |
|  | **cabernet 2a** | 2.04 | 41.64 ± 1.94 | 976.11 ± 1.55 | 1289.1 ± 75.6 |
|  | **cabernet1b** | 1.31 | 45.65 ± 0.62 | 890.62 ± 3.16 | 1813.3 ± 9.6 |
|  | **cabernet 2b** | 2.48 | 69 ± 0.43 | 1157.49 ± 61.93 | 2690.2 ± 30.4 |
| **Sampling day 8** | **cabernet 1a** | 1.18 | 60.58 ± 1.34 | 756.76 ± 17.45 | 1286.9 ± 44.4 |
|  | **cabernet 2a** | 2.75 | 49.15 ± 3.36 | 845.64 ± 14.79 | 1385.1 ± 18.4 |
|  | **cabernet1b** | 1.09 | 50.1 ± 0.02 | 688.23 ± 31.49 | 2113.8 ± 55.4 |
|  | **cabernet 2b** | 2.31 | 77.82 ± 0.06 | 1298.58 ± 21.78 | 2786.9 ± 64.9 |
| **Sampling day 9** | **cabernet 1a** | 0.85 | 61.4 ± 5.53 | 681.67 ± 15.65 | 1228.7 ± 67.7 |
|  | **cabernet 2a** | 1.35 | 63.26 ± 1.32 | 797.67 ± 0.56 | 1295.8 ± 16.7 |
|  | **cabernet1b** | 0.87 | 73.48 ± 2.24 | 622.04 ± 29.57 | 1991.4 ± 80 |
|  | **cabernet 2b** | 2.01 | 105.18 ± 3.07 | 1175.65 ± 3.46 | 2370.2 ± 66.8 |
